# Association between attention deficit hyperactivity disorder and bruxism: A systematic review protocol

**DOI:** 10.1371/journal.pone.0331581

**Published:** 2025-09-11

**Authors:** Anna Carolina Pereira Thimoteo, Júlia Meller Dias de Oliveira, Helena Polmann, Patrícia Pauletto, Cristine Miron Stefani, Graziela De Luca Canto, Lauren Bohner

**Affiliations:** 1 Brazilian Centre for Evidence-Based Research, Federal University of Santa Catarina (UFSC), Florianópolis, Brazil; 2 Department of Dentistry, Federal University of Santa Catarina (UFSC), Florianópolis, Brazil; 3 School of Dentistry, Universidad De Las Américas (UDLA), Quito, Ecuador; 4 Department of Dentistry, University of Brasilia (UnB), Brasília, Brazil; 5 Federal University of São Paulo (Unifesp), São Paulo, Brazil; 6 Department of Oral and Cranio-Maxillofacial Surgery, Hospital University Muenster, Germany; Nigde University: Nigde Omer Halisdemir Universitesi, TÜRKIYE

## Abstract

Attention-deficit/hyperactivity disorder (ADHD) is a neurodevelopmental disorder characterized by the primary symptoms of inattention, disorganization, and/or hyperactivity-impulsivity. Bruxism is a repetitive activity of the chewing muscles characterized by clenching and grinding of the teeth and forceful mandibular movements. Various physiological and psychosocial factors, including attention deficit, depression, stress, and anxiety, have been associated with bruxism. The aim of this systematic review protocol is facilitating the understanding of the systematic review methods and promote transparency in the research. The systematic review will investigate whether there is evidence regarding the association between ADHD and bruxism. The search will be conducted on six databases. Gray literature will be explored through Google Scholar and ProQuest Dissertations & Theses Global. Lists of references of included studies and experts in the field will be consulted. Observational studies that present data comparing a group of individuals with ADHD and a group of individuals without ADHD as well as an analysis of the presence of bruxism will be included. Included studies can detect bruxism based only on a positive self-report (or parental report), clinical inspection, or instrumental assessment. The selection of studies will occur in two phases by two authors. The risk of bias in the included studies will be assessed using JBI tools. A narrative synthesis will be performed. If feasible, a quantitative synthesis will be carried out through pairwise meta-analysis. Odds ratios with 95% confidence intervals will be calculated using the random effects model. This protocol is characterized by its rigorous methodology, which involves an exhaustive search across six databases, as well as gray literature sources, reference lists, and expert consultation, thereby augmenting the comprehensiveness and reliability of the findings. This protocol was registered in PROSPERO under the number CRD42024538099.

## Background

Attention-deficit/hyperactivity disorder (ADHD), characterized by symptoms of inattention, disorganization, and/or hyperactivity-impulsivity [1], often co-occurs with externalizing disorders such as oppositional defiant disorder and childhood conduct disorder. In adulthood, ADHD can negatively affect social, academic, and professional relationships [2].

ADHD is most prevalent in childhood and tends to decrease with age [3]; its global prevalence is 7.2% [4], with 2.5% accounting for adults [3].

Bruxism, which involves repetitive clenching and grinding of teeth by chewing muscles, occurs in two main forms: sleep bruxism (SB) and awake bruxism (AB) [5]. Sleep bruxism, classified as rhythmic or nonrhythmic masticatory muscle activity during sleep, may pose a risk of clinical consequences in healthy individuals [6]. Prevalence studies have shown high heterogeneity according to the detection methods used for SB. The prevalence of SB in adults detected by self-reports is approximately 12.8% (±3.1%) [7], with a decrease to 7.4% when considering SB detected by polysomnography (PSG) [8]. The prevalence of sleep bruxism during sleep declines over time, decreasing from 14% in children to 8% in adults and 3% in patients over 60 years of age [9]. The prevalence of AB varies from 16% to 32% [10].

Bruxism has a multifactorial etiology involving psychosocial, physiological, and behavioral factors, such as respiratory problems, attention deficit, depression, stress, anxiety, and tension [9,11–14].

The diagnosis of bruxism detection is often challenging, both during wakefulness and during sleep [15]. According to Lobbezoo et al., the method of detecting bruxism during wakefulness includes: the use of self-reports (questionnaires), clinical examinations (verification of signs and symptoms), and electromyographic recordings, which are preferably combined with a methodology called ‘ecological momentary assessment. The method of detecting sleep bruxism includes: self-reports (questionnaires), clinical examinations (verification of signs and symptoms), polysomnography (considered the reference standard for the diagnosis of sleep bruxism), and audio/video recordings that can be combined with polysomnography to increase accuracy, as well as electromyography. Therefore, bruxism can be classified as ‘possible bruxism’ when detected by validated questionnaires, ‘probable bruxism’ based on a positive clinical inspection, with or without self-reports (or parental reports), or ‘definite bruxism’ based on positive instrumental evaluations (PSG or electromyography), with or without positive self-reports and/or positive clinical inspection [6].

The hypothesis of an association between ADHD and bruxism was initially proposed by Malki et al. in 2004 [16], motivating further investigations, mainly in children and adolescents. A previous systematic review [17] demonstrated a higher likelihood of bruxism during sleep and wakefulness among children and adolescents with ADHD compared to those without the condition. Since this review focused only on children and adolescents, and the search was conducted in April 2019, our objective was to conduct a new systematic review with the justification of investigating the association between ADHD and bruxism without age restrictions. This is based on several key factors: a comprehensive understanding across different age groups is necessary, as they may exhibit distinct manifestations throughout life; bruxism in childhood may be influenced by factors such as sleep disorders and anxiety, while in adulthood, factors like stress or medication use may be more prominent. By not restricting the review to a specific age group, we can gather broader evidence, allowing for a better understanding of how wide-ranging and consistent the ADHD-bruxism association can be. Limiting the analysis to specific age groups may introduce selection bias, as studies focused solely on children or adults may miss important data or patterns that emerge when considering the entire population. Conducting a systematic review encompassing all ages allows researchers to identify gaps in the literature related to specific age groups.

## Methods

### Protocol and registration

This protocol was developed according to the Preferred Reporting Items for Systematic Reviews and Meta-Analyses Protocols (PRISMA-P) [18] (S1, Appendix 1). It has been registered in the International Prospective Register of Systematic Reviews (PROSPERO) under the number CRD42024538099.

### Ethics Statement

This study did not require ethical approval, as it is a protocol for a systematic review. The research will be based exclusively on previously published data, and no primary data collection or involvement of human participants will be conducted.

### Research question

This systematic review seeks to answer the following question: “Is there an association between ADHD and bruxism?”. The research question is based on the PECOS acronym (Table 1).

**Table 1:** Research question based on the PECO acronym.

P (Population)	Children, adolescents, and adults
E (Exposure)	Attention Deficit Hyperactivity Disorder (ADHD)
C (Comparison)	Individuals without ADHD
O (Outcomes)	Occurrence of bruxism (awake and/or sleep)
S (Type of study)	Observational studies

### Eligibility criteria

#### Population.

Children (up to 12 years old), adolescents (13–18 years old), and adults (>18 years old), irrespective of sex, racial background, ethnic origin, and environment.

#### Exposure.

*Attention Deficit Hyperactivity Disorder*. Studies in which the diagnosis of ADHD was made using any edition of the Diagnostic and Statistical Manual of Mental Disorders (DSM) [2,19,20] diagnostic criteria, structured interviews, neurological assessments, validated questionnaires, or other types of objective validated methods will be included.

#### Comparator.

Individuals without ADHD.

### Outcomes

*Bruxism.* The presence of bruxism will be determined according to the criteria proposed by the International Consensus [6]. Studies will be accepted regardless of whether they are about sleep/awake bruxism, possible sleep/awake bruxism based only on a positive self-report (or parent report), probable sleep/awake bruxism based on a positive clinical inspection, with or without self-report (or parental report), definitive sleep/awake bruxism based on positive instrumental assessment (PSG or electromyography), with or without positive self-report and/or positive clinical inspection.

### Types of studies to be included

Observational studies (cross-sectional, cohort, and case-control) comparing the occurrence of bruxism (awake and/or sleep) in a group of individuals with ADHD with that in a group of individuals without ADHD. Studies carried out in any setting, such as hospitals, clinics, sleep laboratories, or population-based studies, will be considered.

### Exclusion criteria

1. Studies involving specific groups, such as people with congenital anomalies, a history of bipolar disorder, psychosis, anxiety disorder, Tourette’s disorder, autism, a history of substance abuse, or other neurological conditions associated with marked impairment of attention;
2. Studies that did not specify the method for detecting bruxism and/or ADHD; did not have the data from the bruxism group; did not have a control group; or did not evaluate the association between bruxism and ADHD;
3. Reviews, letters, editorials, books, conference summaries, case reports, opinion articles and guidelines;
4. Full text not available, even after attempting to contact the corresponding authors (three attempts over a period of three weeks).

### Information sources and search strategy

A preliminary search strategy was formulated and refined with the assistance of a health science librarian. Subsequently, a comprehensive search strategy was developed for each database (S2 Appendix 2). A systematic search will be conducted in the following databases from inception to present: Embase, MEDLINE (via PubMed), LILACS, LIVIVO, Scopus, and Web of Science. Gray literature will be explored through Google Scholar and ProQuest Dissertations & Theses Global. Additionally, reference lists of included studies and input from experts in the field will be consulted. We will consider experts, the authors of more than four studies included in our review. Additionally, we will read the studies included in the previous systematic review [17] on this topic to verify whether they fit our eligibility criteria.

### Data management

The files from each database will be exported to EndNote (EndNote-Web^TM^, Clarivate^TM^, Jersey, USA) [21]. They will be merged, and duplicate references will be identified and excluded. After that, a single file will be exported to (Rayyan®, Qatar Computing Research Institute, Data Analytics, Doha, Qatar) [22], where the selection process based on the eligibility criteria will be carried out independently by two authors (APCT, JMDO).

### Selection process

First, two authors (ACPT, JMDO) will read the abstracts of five studies and perform a preliminary pilot test according to the eligibility criteria as a way to calibrate the authors. After the pilot test, a two-phase process will be executed and overseen by the same two independent authors (ACPT, JMDO). In phase 1, the two authors will autonomously screen the titles and abstracts of all identified references. Studies failing to meet the eligibility criteria will be excluded. During phase 2, the same two authors will apply the eligibility criteria to the full texts of the studies. In cases of disagreement concerning article inclusion or exclusion, a third author (HP) will be consulted, and consensus will be reached through discussion. Online doc translator software will be used to translate abstracts and articles not written in English, Portuguese, or Spanish [23]. The study selection process will be meticulously documented to facilitate the completion of the flow diagram as per PRISMA 2020 guidelines [24]. Excluded studies and their rationales for exclusion will be compiled in a table labeled “Characteristics of excluded studies” and presented in the article as a supplementary file.

### Data collection process

An initial data extraction template will be created using Microsoft Excel version 4.7.7 (Microsoft Office 365, Microsoft® Corporation, Redmond, Washington, USA) [25]. The variables collected will focus on capturing specific details related to the PECOS framework.

Data extraction from the included studies will be performed by one author (ACPT) and cross-checked by the second author (JMDO) using the data extraction tool developed by the authors. Discrepancies will be addressed through discussion between the two authors (ACPT, JMDO). If disagreements persist, a third author (HP) will be consulted. The extracted data will be presented in the article within a table detailing the characteristics of the included studies. In cases involving duplicate publications or primary studies with multiple reports, all available data will be collected and incorporated into a comprehensive dataset aggregating information from all known publications. In instances where essential data are missing, the corresponding authors of the original studies will be contacted by email to request missing data. Up to three emails will be sent, with an interval of 15 days between them. If the data cannot be obtained, they will be considered unavailable. Only available data will be subjected to analysis.

### Data items

The following data will be collected from the included studies: study characteristics (author, country, year, setting, study design); participant characteristics (number, age, sex); criteria for ADHD diagnosis; criteria for bruxism diagnosis; description of ADHD and bruxism; (type of sleep/awake, primary/secondary); number of bruxism events in both groups, use of medication; results; conclusions; funding; and conflicts of interest.

### Risk of bias in individual studies

Two independent authors (ACPT, JMDO) will evaluate the risk of bias in the included studies using JBI’s critical appraisal tools, according to the design of the included studies [26] (Table 2). The selection of tools will align with the study types included. Before tool application, the authors will deliberate on the choice of tool and establish evaluation parameters. Subsequently, a calibration exercise will be conducted between the evaluators by using two included articles. Any discrepancies between the authors will be resolved through discussion and consultation with a third author (HP) if necessary. Figures regarding the risk of bias will be created on the online tool *robvi*s (Risk-Of-Bias VISualization) (National Institute for Health Research) [27].

**Table 2:** Risk of bias tools [26].

Study design	Tool
Cross-sectional	JBI Checklist for analytical cross-sectional studies
Case‒control	JBI Checklist for case-control studies
Cohort	JBI Checklist for cohort studies

### Data synthesis

A narrative synthesis will present the results of each included study. If quantitative synthesis is possible, a pairwise meta-analysis of associations will be performed using RevMan Web [28]. Odds ratios with 95% confidence intervals will be calculated using a random-effects model for the overall meta-analysis and subgroup analyses.

### Heterogeneity

Statistical heterogeneity will be evaluated among the results of different trials using the chi-square (Chi^2^) test, with significance defined as P < 0.1. The I^2^ statistic will be utilized to assess heterogeneity within studies in each analysis. We will consider evidence of heterogeneity if the Chi2 test p-value is < 0.10 and the I^2^ test result is 50% or above. In this case, possible reasons for heterogeneity will be explored through subgroups or sensitivity analysis. Furthermore, heterogeneity will be examined by visually inspecting the overlap of confidence intervals (CIs) and may be explored in subgroup analyses if applicable. We will investigate the influence of studies with a high risk of bias on the overall effect estimate by excluding studies with these characteristics from each meta-analysis (sensitivity analysis) [29].

### Subgroup analysis

Subgroups are planned by age (children, adolescents, or adults), type of bruxism (SB/AB and possible/probable/definitive), and type of sample (convenience and population-based). Eventually, depending on the data found in the included studies, other exploratory subgroup analyses can be carried on.

### Sensitivity analysis

The robustness of the findings will be assessed in a sensitivity analysis by removing studies with a high risk of bias from the meta-analysis. Other characteristics of the data will also be explored in sensitivity analyses, such as the impact of the studies with convenience samples and studies with a small sample size.

### Assessment of reporting bias

To assess evidence of selective nonreporting of results, each study included in the main meta-analyses will be evaluated by comparing the reported information in the paper with other available sources, such as trial registry entries, study protocols, or statistical analysis plans, to identify any outcomes without available results in the report. This information will be collected during data extraction. In cases where no other information source is available, methods and results sections will be cross-checked to identify potential missing or incompletely reported results. Publication bias will be assessed through funnel plots and Egger’s test if ten or more articles are included [30].

### Confidence in cumulative evidence

Since there is currently no guidance from the GRADE Working Group for systematic reviews of association and considering that the “Requirements for claiming the use of GRADE” advise against modifications and adaptations of the GRADE approach [31], the certainty of the evidence will not be assessed.

## Discussion

This is a protocol for a systematic review that aims to investigate the association between ADHD and bruxism. The topic has aroused interest in the literature due to the increasing incidence of both conditions around the world in different age groups [10].

A previous systematic review indicates that children and adolescents diagnosed with ADHD have a higher chance of developing both SB and AB compared to controls without ADHD. The authors included 32 studies revealing that the proportion of children with bruxism in the ADHD group was approximately 31%. ADHD was associated with increased odds of bruxism (sleep and awake) of 2.94, suggesting nearly three times the likelihood of developing this condition [17].

The literature suggests that the pathophysiology of bruxism can involve both peripheral and central mechanisms [32]. Bruxism may be associated with transient arousals during non-REM sleep, during which excitatory neurotransmitters momentarily surpass inhibitory mechanisms, resulting in the activation of jaw muscles [32]. Besides that, it is known that bruxism can be associated with psychosocial factors, stress, anxiety, and genetic predisposition [13,14,33,34]. Other associated factors are the continuous use of medication, such duloxetine, paroxetine, venlafaxine, barbiturates and methylphenidate might be associated with SB [35] and drug abuse, caffeine, smoking, and alcohol intake [36].

The possible association between ADHD and bruxism can be attributed to several factors. Individuals with ADHD often experience higher levels of stress and anxiety, which can contribute to development of bruxism. Also, some medications used in the ADHD treatment have been linked to an increase of secondary bruxism [16].

Interesting to note that the prevalence of both conditions decreases with age. ADHD is most prevalent in childhood and tends to decrease with age [3]; its global prevalence is 7.2% [4], with 2.5% accounting for adults [3]. The prevalence of sleep bruxism declines over time, being 14% in children, 8% in adults, and 3% in patients over 60 years of age [9].

Although the hypothesis of an association between bruxism and ADHD was made by Malk et al. [16] more than 20 years ago, the possible association between bruxism and ADHD is not well established in the literature.

A differentiated approach in the discussion about the relationship between ADHD and bruxism will benefit both health professionals and patients, as well as policymakers, allowing them to make informed decisions regarding this association. An integrated interdisciplinary treatment plan can, therefore, improve the quality of life for patients. Moreover, we hope to identify gaps in the literature and suggest directions for future research, providing insights that can enhance the clinical and therapeutic management of these individuals.

The strengths of our protocol will be its rigorous methodology, which involves an exhaustive search across six databases, as well as gray literature sources, reference lists, and consultation with experts, thereby augmenting the comprehensiveness and reliability of the findings. Also, to maintain study integrity and mitigate selection bias, the review will employ two blinded reviewers assisted by a third reviewer in the decision-making process. This comprehensive analysis aims to yield a profound understanding and valuable insights for dentists and psychiatrists, facilitating the diagnosis of both conditions and the formulation of treatment plans.

The main challenge will likely be the different methods used to detect bruxism in the included studies.

We aim to report the systematic review as an article and publish it in a recognized journal. Additionally, we intend to present the results in national and international meetings. We will make all our findings available without restriction. If it is not possible to present them in full in the article, they will be deposited in a freely accessible repository.

If there are important protocol amendments we plan to document them by adding this information in the systematic review article and the protocol registered in PROSPERO.

## Supporting information

S1 Appendix PRISMA-P Checklist. (DOCX)

S2 Appendix Search Strategies. (DOCX)

## Abbreviations

AB: Awake bruxism
ADHD: Attention deficit hyperactivity disorder
CIs: Confidence intervals
DSM: Diagnostic and Statistical Manual of Mental Disorders
GRADE: Grading of Recommendations Assessment, Development and Evaluation
LILACS: Literatura Latinoamericana y del Caribe en Ciencias de la Salud
PRISMA-P: Preferred Reporting Items for Systematic Reviews and Meta-Analyses Protocols
PROSPERO: International prospective register of systematic reviews
PECOS: Acronym – P: Population; E- Exposure; C-Comparison; O-Outcomes; S- study design
PRISMA: Preferred Reporting Items for Systematic Reviews
PSG: Polysomnography
SB: Sleep bruxism
